# Quality detection and grading of peach fruit based on image processing method and neural networks in agricultural industry

**DOI:** 10.3389/fpls.2024.1415095

**Published:** 2024-09-20

**Authors:** Dan Luo, Rong Luo, Jie Cheng, Xin Liu

**Affiliations:** ^1^ College of Intelligence and Computing, Tianjin Ren’ai University, Tianjin, Tianjin, China; ^2^ School of Intelligent Computing Engineering, Changji University, Changji, Xinjian, China

**Keywords:** machine vision, image processing, clustering, classification, artificial neural network

## Abstract

The grading of products is important in many ways. One of the important activities after harvesting agricultural products is product grading based on shape and color dimensions. This activity in the agricultural transformation industries, Bas Controller, improves various processes on fruits and vegetables with the same dimensions, which improves the storage conditions of the product, creates added value for the farmer, and gives the consumer the power to choose. The main focus of this study is the application of image processing in the field of identification and classification of fruits. It is an application that has received much less attention than other applications of image processing. The proposed systems presented in this article, are software solutions based on image processing techniques, including histogram matching techniques, for detection, Sable edge detection algorithms, Private edge and Kenny edge, Otsu threshold limit, and clustering method It is an automatic mean and classification of different degrees of fruit. In addition, it has been mentioned more about the examination and description of product grading and clustering methods, that by using the proposed application hardware and its connection with the software, a big step can be taken in product quality grading. This method can be used in product classification and packaging. The accuracy rate for peaches, lemons, apples, and tomatoes is 94.58%, 88.23%, 70%, and 93.33%, respectively. The best accuracy for all 20 sample levels is for peach fruit.

## 1 Introduction

In today’s world, with the existing concerns about the lack of resources, it is necessary to move agriculture towards sustainable agriculture, for example, biological agriculture, or intensive agriculture, for example, industrial agriculture, so that we can meet the needs in the future. Agriculture includes a wide range of expertise and techniques, including ways to expand land suitable for plant cultivation, digging canals, and different forms of irrigation. Modern agriculture, plant breeding, pesticides, stagnant pests, automatic sorting, and technological advances greatly increase crop yields (Bajwa et al., 2015; Baker et al., 2017). One of the important applications of machine vision is the inspection of factory output products and their quality control. Technological progress in image processing has opened up a wide range of machine vision applications in agriculture, and the development of powerful microcomputers and special software has led to the applicability of image processing to inspect agricultural products and fruits, especially in the field of quality control and classification. has been Today, many agricultural products sorting systems are used to separate fruits and products according to color, shape, size, weight, degree of damage, bursting and crushing, etc (Silva et al., 2018; Benbarrad et al., 2021; Smith et al., 2021). They use the machine vision method and image processing capabilities, in these systems, images of the products that are moving on the conveyor system are taken by a camera and transferred to the computer for processing and the required data from it. is extracted, then based on the obtained information, orders are issued to activate or deactivate a mechanical part in order to remove the product from the main path or allow it to pass through the path (Eissa et al., 2012; Nerakae et al., 2016; Nouri-Ahmadabadi et al., 2017; Baygin et al., 2018; Benbarrad et al., 2021).

The two actions of sorting and grading have been discussed in many industries. Using machine vision technology is the best solution for this because it has the lowest cost, error, and the highest accuracy and quality compared to mechanical systems (Thong et al., 2019; Lin et al., 2020; Zhang et al., 2020). One of the fields where there is an urgent need for the presence of sorting and grading systems based on machine vision is agricultural fields. With the help of machine vision technology, agricultural products are separated and graded with better accuracy and quality and at a lower cost (Tian et al., 2014; Taghadomi-Saberi and Hemmat, 2015; Sohail et al., 2021; Wang et al., 2022). Appearance (external) characteristics cannot be a good criterion for checking the quality of some products, some agricultural products such as apples or oranges have shriveled or shriveled internal tissue due to diseases or pests and lack of water, and the internal quality of these products has decreased, and as a result, the need It is used to separate the healthy product or to separate the product in different groups. It is a quick, accurate, and non-destructive method for grading apples based on their specific gravity using a weight sensor and machine vision. Some dimensional components of potato band, length, width, thickness, area, and volume in several different figures are calculated using the image processing technique. A comparison of the values obtained with the values measured in the laboratory shows that the use of machine vision has brought satisfactory results and can also help researchers and managers in this field to achieve precise agricultural goals. Today, many fruits are traditionally and visually trained and inspected and evaluated by the operator, today’s world is moving towards the mechanization of the industry, and the need for intelligent and automatic systems in the field of inspection, and quality control of all kinds of fruits is quite noticeable. In this method, all work steps are controlled automatically and two important parameters of image processing and machine vision are used (Lins et al., 2020; David et al., 2021; Wang et al., 2022).

Among the advantages of using such systems are that are able to detect the level of quality, we can mention more speed, cost, and less error. The image processing technique system has been used as a new, accurate and fast method to measure the dimensions of small seeds and mucilage such as basil and myrtle seeds (Lust, 2014; Barradas and de Holanda e Silva, 2021; Gorjian et al., 2022). In order to check the accuracy of this method, its results are compared with the data obtained from measuring with a micrometer and the correlation coefficient between the two methods is obtained. The research is carried out in order to provide an algorithm for detecting the infancy of watermelon fruit. First, the physical characteristics of watermelon, including mass, volume, dimensions, and the apparent mass of watermelon in three sizes, large, medium, and small, are measured. Then, the relationships between the above characteristics are investigated for the standard state of the fruit and malformed varieties, and the images taken of the fruit are calibrated. Using the obtained relationships, a hybrid detection algorithm is presented. The results showed that the ratio of the length to the width of the image and the ratio of the area of ​​the image of the fruit in the two-dimensional state of the pistachio background can be used as a suitable criterion for detecting malformation. The image processing technique is used as a new and fast method for classification based on apple quality, which is processed using the software. The main goal of the project is to eliminate human labor from the process of direct visual inspection of packaging, which will greatly help farmers (Jain, 2021). The core of this project is a computer device and an advanced recognition and description program, which processes the images received from the camera by comparing the images, good and bad apples are identified, and by comparing the obtained parameters and putting the results together. From the processing performed on the image, quality apples can be recognized (Song et al., 2020; Li et al., 2021).

Since agriculture dates back to ten thousand years ago, it has a wide range all over the world. Agriculture 2016 (Andersen et al., 2016). Different methods in agriculture such as irrigation, crop rotation, fertilizers, and pesticides are developed long ago, but huge steps have been taken in the last century is removed. In early societies, people whose agricultural production exceeded the needs of their families are able to attract other people to their side. Some historians believe that the development of agriculture led to the emergence of civilizations. Agriculture is the production of food and goods through agriculture, forestry, and animal husbandry. One of the problems that cause the quality of agricultural products to decline is the lack of water and improper nutrition of the products. Agricultural products are one of the main sources of human nutrition. In modern agriculture, various methods are used to increase the yield of these products. Agricultural experts identify low-quality products with the naked eye. But this work, in addition to requiring a lot of money and manpower, has had low efficiency in identification and classification. Especially when the agricultural land is large, the cost increases. Today, survey methods have become unreliable. Every day, the demand for using new technologies increases, among the fields that can help the agricultural industry are artificial intelligence technologies. The applications of artificial intelligence in agriculture include a variety of cases, including mapping of the earth’s surface, water and agricultural conditions of the land, the condition of pastures, and extraction of agricultural information. Also, one of the most widely used advantages of artificial intelligence in agriculture is the automation of systems for planting, keeping, harvesting, product supply, and packaging. Image processing is one of the important branches of this technology, which is aimed at helping different fields, including the agricultural industry. Determining the level of quality is another service of new artificial intelligence technologies for agriculture. The use of algorithms that are mentioned in the computational methods of artificial intelligence. It has a very high ability to process the captured image of agricultural products for grading. Artificial neural networks are one of these new computing methods. Because this method has brought advantages such as increased speed and greater accuracy and reduced costs with the automation of the diagnosis and investigations process (Dimililer and Zarrouk, 2017; Deng et al., 2020; Kasinathan and Uyyala, 2021; Ngugi et al., 2021). The recorded images consist of many small squares (pixels). Each pixel has a multi-digit number that indicates the amount of light (color) of that pixel. Image accuracy depends on the number of pixels, and the more bits the image has, the brighter and clearer the image will be (Ariff et al., 2020; Pustokhina et al., 2020; Yousif et al., 2020; Chen and Zong, 2021).

The increase in population around the world requires a satisfactory level of crop production with a decrease in the amount of agricultural land. In the past few years, significant results have been achieved in various sectors of agriculture. Machine vision ensures increased productivity using an automated, non-destructive and cost-effective method, these achievements are achieved with machine learning techniques in a landscape approach that deals with color, shape, texture, and spectral analysis of object images. They are integrated. Due to the wide range of machine learning applications, this review only describes statistical machine learning technologies with machine vision systems in agriculture. Two types of learning techniques such as supervised and unsupervised learning have been used for agriculture (Rehman et al., 2019; Kakani et al., 2020; Sharma et al., 2020; Meshram et al., 2021). Image processing is a branch of computer science that refers to the processing of digital signals received by digital cameras or similar devices. Image processing is divided into machine vision and image processing. Image processing is more related to improving images, but machine vision includes methods of understanding images. In the early 1960s, NASA’s Ranger spaceship began sending fuzzy television images of the moon’s surface to Earth and extracting image details to find a landing site for the Apollo spacecraft required applying decisions to the images, thus beginning the specialized field of digital image processing. Like all other technologies, it quickly found many uses. Since 1964, the topic of image processing has grown so much that, in addition to the space research program, image processing techniques are now used in many cases, including medicine, archeology, automotive industry, food industry, and packaging. The application of image processing has also made significant progress in agriculture in recent years. Image processing includes imaging steps; Preprocessing is feature extraction and registration (Vibhute and Bodhe, 2012; Singh et al., 2020). In today’s agriculture, it is important to choose methods that save time and money. Also, in choosing a method to check the properties and conditions of the product, one should use a method that causes less damage to the product. Image processing and machine vision are among these things, which have found special use in various sciences, including in agriculture, this technology has found a significant place so that it has been introduced in its various stages and led to the construction and production of many machines and robots in the field. The machine has become vision (Chlingaryan et al., 2018; Darwin et al., 2021). technologies, the discovery of new agricultural methods as well as new techniques to prevent crop reduction due to pests, natural disasters, and drought in general, and fruit quality inspection is done by human experts (Gondal and Khan, 2015; Kapoor et al., 2016; Thenmozhi and Reddy, 2017). This manual classification by visual inspection is labor-intensive, time-consuming, and difficult, and may suffer from the problem of inconsistency and inaccuracy in judgment by different people. With the advent of high-speed and high-precision vision technologies, the automation of the grading process is expected to reduce labor costs, and improve the efficiency and accuracy of the sorting process.

Due to the necessity and importance of the mechanization of agricultural systems and the help of image processing, many research and public works have been done on this issue. C.S. Nandy et al. have made a classification system based on the color and ripeness of mango fruit using an image processing system and a mechanical design. In this research, 750 samples from 5 different regions and a 10-megapixel CCD camera were used to investigate the differences (Nandi et al., 2014). Yahua Zhang et al. 160 apples have been examined to detect defective apples. Due to the similarity of the defective part with the healthy part, especially at the edges of the apple skin, for machine vision detection with automatic light correction, the sample part number and the weight of the cluster Relevance Vector Machine (RVM) are combined (Mizushima and Lu, 2013). A new automatic method for mango sorting based on computer vision algorithms is presented by Pauli and Sankar. The use of this system replaces the existing manual methods of sorting in India, the system for the Alfano mango model which has led to the development of exports in India. The developed system is able to sort the Alphonso mango with 83.3% accuracy and recognize the defective skin with minimal area (Pauly and Sankar, 2015). The purpose of this article is to deal with the application of image processing and machine vision in the stage of picking and categorizing and grading the quality of the product before entering the market and pricing the product accurately. Ministry of Jihad Agriculture and gardeners can benefit from the results of the research, which can systematically assess the quality and grading of their products after harvest. Most importantly, the buyers of the product, i.e., ordinary people, will also benefit from this research. Because the quality of the product and its grading are of great importance for public use. Therefore, this survey can be used by agricultural experts to grade the quality of products and price that products.

## 2 Proposed method

Our goal of this proposed plan is to build a system with high efficiency and small in terms of structure and price for classifying agricultural products. Also, the proposed plans can be a scaled-down example of an industrial plan that can be changed according to the conditions and facilities. As the grading and classification of agricultural products can have a direct effect on the country’s exports, increasing accuracy in classification is very important to attract customers. Therefore, it is better to check the things that affect the quality, among these things, the color, size, and weight of the products, each of the characteristics can be important based on the type of fruit alone with a combination. To increase the accuracy, all three characteristics will be checked and calculated in this proposed design.

MATLAB software is a strong and advanced program that has many libraries in the field of image processing. According to the definition of the goal and the action to be performed, suitable blocks can be used, in the definition of the proposed method, it is necessary that the color and size be measured by the program. The blocks used are placed in combination and each section is connected to the next section. Weight measurement is done by the weight sensor in the hardware section. Since we need the weight to be considered in the measurement, the measured value is entered into the program. Finally, it is combined with the amount of color and size. Based on the combination of the amount of fruit, it is placed in the appropriate category. Considering the placed handle, one of the control commands related to the separator is activated.

### 2.1 Color analysis

In MATLAB software, it takes the image from the camera (webcam) in real time using the function cam = videoinput (‘winvideo’). At the same time, the front part of the image can be displayed in raw black and white. Using the output wire, the image is sent to the next section for analyzing the characteristics. **Supplementary Figure S1** shows the image of the camera.

To measure color, as mentioned in the previous chapter, red, green, and blue colors are separated and their values are measured separately. The method of getting the color is that you can get the amount of color from a pre-defined point. The steps of fruit color calculation are shown in **Supplementary Figure S2**. But the experiments it shows that taking the color from several points and taking the average of all three colors red, green, and blue reduces the amount of error. Therefore, in this method, n points (minimum 5 and maximum 25) are used to measure color.

**Supplementary Figure S3** shows the range considered in peach fruit. Points are considered within the specified range.

In this method, a range is defined according to the size of the fruit. The number of points in this range is selected, finally, the average color and the average values ​​of red, green, and blue are obtained. The input image in this section has two RGB (32) and HSL (32) modes.

### 2.2 Measurement

**Figure 1:**
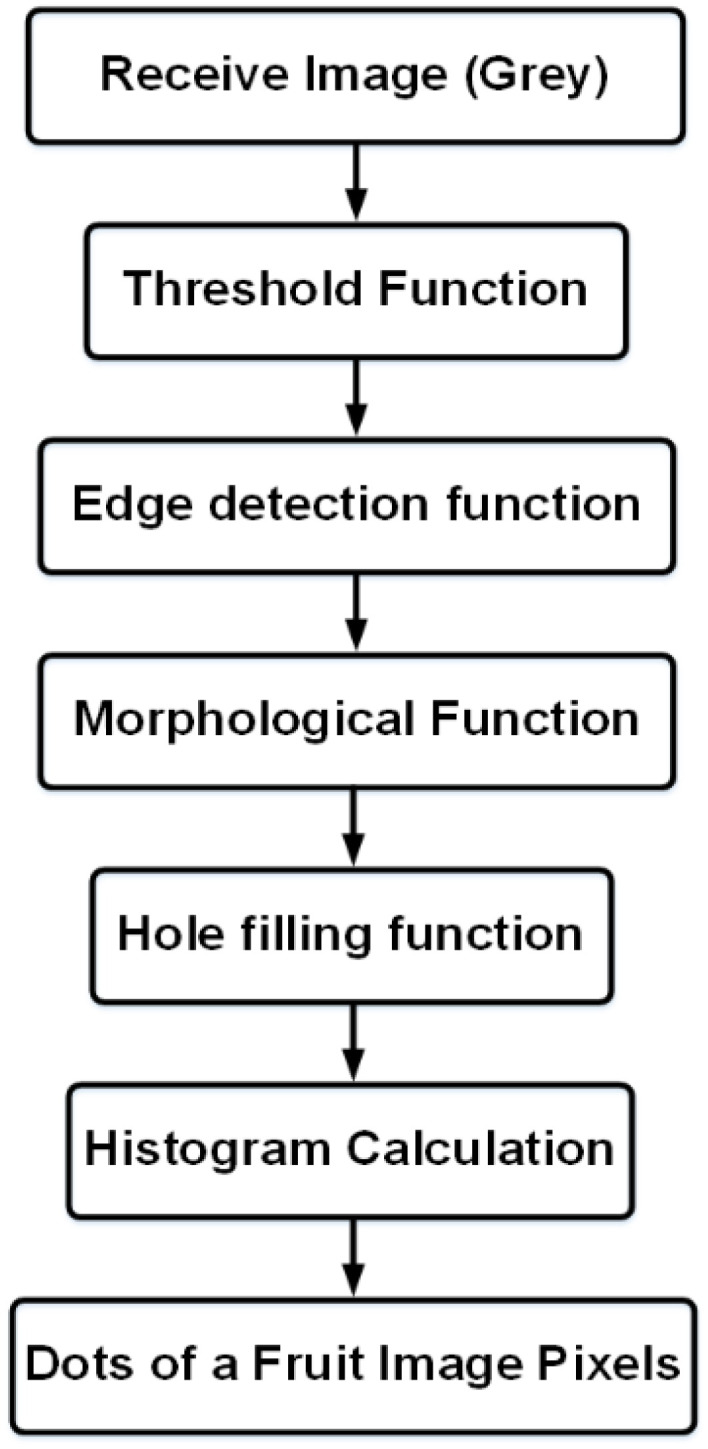
Functions used to calculate the size.

In this method, the initial image is converted into a gray image, then the background is separated from the fruit using the Threshold function. Using the *imfill* function, the surrounding edges are separated after the edge. The functions used to calculate the size are shown in **Figure 1**.

**Figure 2:**
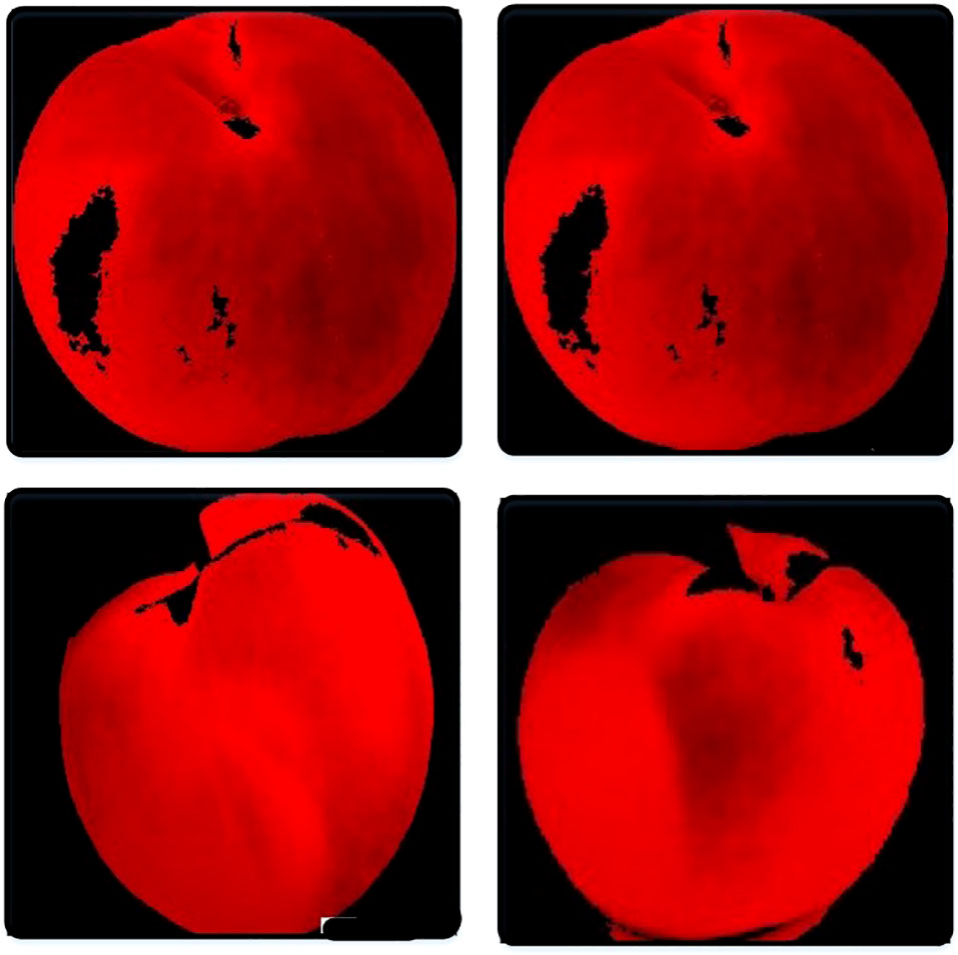
Shows the color analysis sample.

**Supplementary Figure S4** shows the measured sample and **Figure 2** shows the color analysis sample.

Due to the gray color of the image, which can have a wide spectrum based on the light, the Morphology function is used to equalize the gray color. Finally, after equating all the points with the adjacent point (*nohole* function), the number of pixels in areas 0 and 1 is measured. The number of points 1 displays the number of pixels of the fruit. In this proposed system, it can calculate the amount of fruit size by several methods. According to the tests and evidence, the following method seems more accurate.

### 2.3 Combination of specifications

To calculate the quality of all three parameters, color, size, and weight are important, so all three should be considered at the same time, although in some products the color changes may not be very visible. Most of the size and weight determine the level of quality. In this situation, it is possible to give a coefficient for each of the characteristics. In this plan, the importance level is considered the same, or the Q value or the quality of the proportional quality of each category is calculated, with the help of this value, the degree and the error rate can be obtained. The value for Q can be taken as the minimum and the background. In this situation, the minimum and background values ​​of the minimum and maximum values of Q of each degree are considered. If the value of Q is within the considered range, one of the four outputs will be activated according to the quality of the fruit.


(1) $$Max=\overset{-}{X}+Var(x)$$

(2) $$Min=\overset{-}{X}+Var(x)$$

(3) $$\overset{-}{X}=\frac{\sum xi}{n}$$

(4) $$Var=\frac{\sum_{i=1}^{n}{\left(x_{i}-x\right)}^{2}}{n}$$

(5) $$d_{R}^{2}={\left(\frac{R_{m}-R_{x}}{R_{v}}\right)}^{2}+{\left(\frac{B_{m}-B_{x}}{B_{v}}\right)}^{2}+{\left(\frac{G_{m}-G_{x}}{G_{v}}\right)}^{2}$$

(6) $$d_{S}^{2}={\left(\frac{S_{m}-S_{x}}{S_{v}}\right)}^{2}$$

(7) $$d_{w}^{2}={\left(\frac{W_{m}-W_{x}}{W_{v}}\right)}^{2}$$

(8) $$Q_{i}=\frac{\left(e^{-d_{R}}+e^{-d_{W}}+e^{-d_{S}}\right)}{3}$$

Based on the result of the specification section, one of the four modes of the separating motors is activated. The results of the previous section are entered into the command section after the matching mode is detected and based on the results of the two considered motors according to the program written in Code vision at four different angles. In addition to the two separator motors, there is a conveyor motor with a control button in the program. How the motors work will be explained in the hardware section. The second part of the control commands is related to the movement of the motor, moving the product from the weight sensor to the conveyor belt. The voice command is that after the sample is placed on the sensor, the motor will move forward after two seconds. In this study, clusters are created based on three different color intensities in the image including background, shadow, and fruit. The average is calculated from Equation 9.


(9) $$\mu =\frac{\sum_{i=1}^{n}P_{i}}{n}$$

where p is the value of each pixel, and n is the number of pixels. Variance is a simple way to measure the spread between data. A low variance indicates that the data are close to the mean. A high variance indicates that the data is spread over an extensive range. It is calculated using Equation 10.


(10) $$\sigma^{2}=\frac{\sum_{i=1}^{n}{\left(P_{i}-\mu \right)}^{2}}{n}$$

where *n* is the number of pixels, *p* is the pixel value, and *µ* is the average. The red, green, and blue components of the feature-finding algorithm are calculated from Equation 11.


(11) $$r=\frac{R}{R+G+B},g=\frac{G}{R+G+B},b=\frac{B}{R+G+B}$$

Finding the color hue is calculated by the relations of Equation 12.


(12) den=((r−g)2+(r−b)×(g−b)), num=0.5×((r−g)+(r−b))theta=acos(num(den+eps)),theta(b>g)=2×pi−theta(b>g),Hue(H)={0ifS=0theta(2×pi)ifS≠0

The saturation component is also calculated using Equation 13.


(13) $$\begin{array}{l} \min=\min imum\;of\;(R,G,B),\\ Saturation(S)=1-\frac{\min}{Intensity\;(I)} \end{array}$$

And the intensity component of the images is calculated using Equation 14.


(14) $$Intensity\;(I)=\frac{R+G+B}{3}$$

## 3 Evaluation parameters and dataset

Classification is done with the back-propagation neural network, whose superiority is its simplicity, adaptability, and accuracy, and it is used to classify patterns. The extracted color features are sent to the neural network for training and testing the proposed method. Training and target vectors are created. The training vector includes the features and the target vector includes the classes vector that belongs to each component of the training vector. MATLAB neural network toolbox is used for implementation.

### 3.1 Evaluation parameters

Sensitivity, specificity, and accuracy parameters have been used for evaluation. Sensitivity: shows the proportion of correct positive diagnoses. The higher this value means, the fewer false positive results are returned, and it is calculated from Equation 15.


(15) $$Sensitivity=\frac{TP}{TP+FN}\times 100$$

TP (True Positive): Ripe fruit can be recognized as ripe. FP (False Positive): unripe fruit, wrongly detected as ripe. TN (True Negative): unripe fruit, correctly diagnosed as unripe. FN (False Negative): Ripe fruit, wrongly diagnosed as unripe. Feature: Negative items that have been correctly diagnosed. and it is obtained from Equation 16.


(16) $$Specificity=\frac{TN}{TN+FP}\times 100$$

Accuracy: The diagnostic items are correct. Both true positive diagnosis cases and true negative diagnosis cases are calculated from Equation 17.


(17) $$Accuracy=\frac{TP+TN}{TP+TN+FP+FN}\times 100$$

The proposed method for products with external defects outside the quality standards. Therefore, in the implementation and sampling section, products with apparent defects have been removed. It is designed to measure the color, size and weight of products for automatic sorting. This method has two parts, software and hardware, both parts are completely related to each other. Therefore, there are many correct connections between these two parts. In case of problems in any of the parts, the wrong result will be obtained. In this method, it is possible to change the goals, for example, the program can be designed in such a way that stains or defects are also considered, but it is assumed.

### 3.2 Dataset

The main objective is to check the color, size, and weight of peach fruit. By further investigating the conditions of other products, it is concluded that this test can be performed in the case of tomatoes, tangerines, etc. **Table 1** shows the grading of the used samples of apple, tomato, lemon, and peach fruits.

**Table 1:** Grading of used samples.

-	Grade 1			Grade 2			Grade 3			Grade 4		
Apple	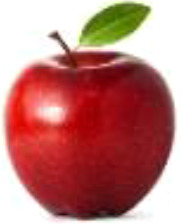	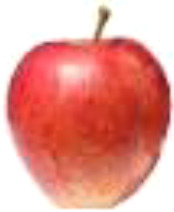	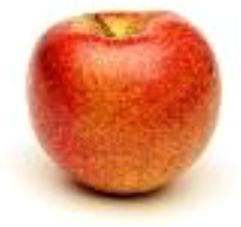	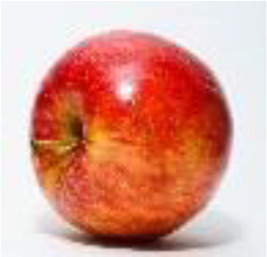	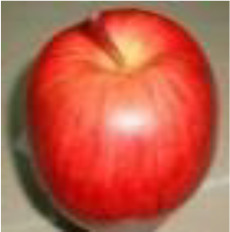	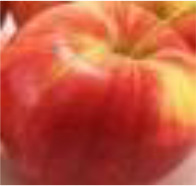	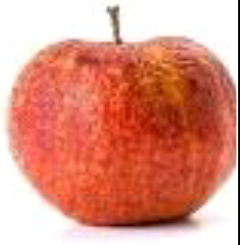	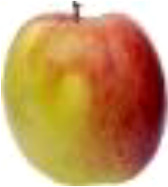	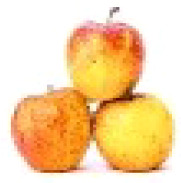	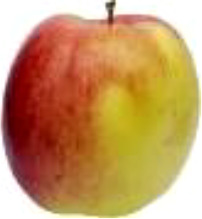	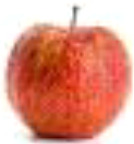	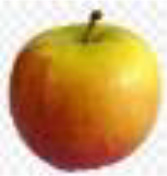
Tomato	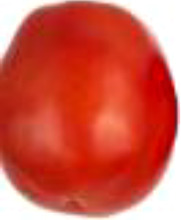	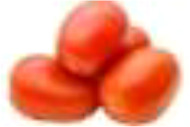	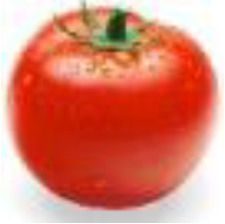	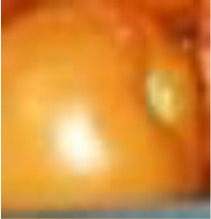	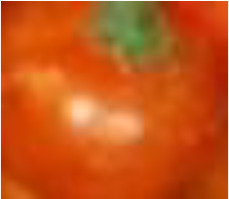	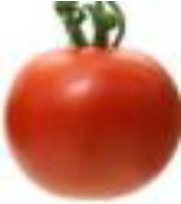	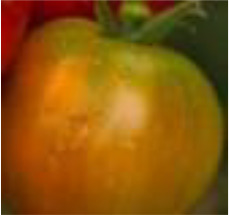	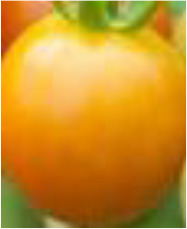	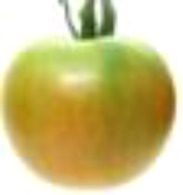	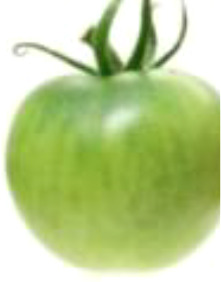	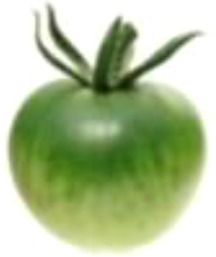	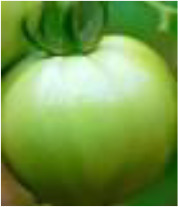
Lemon	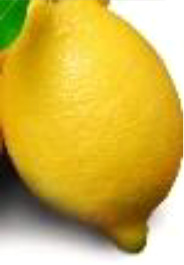	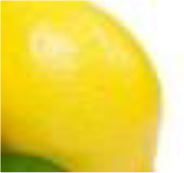	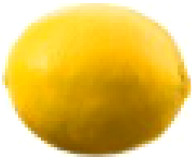	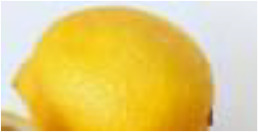	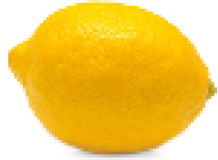	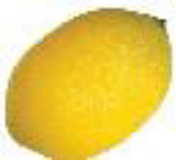	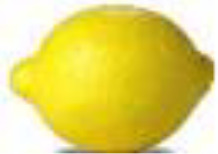	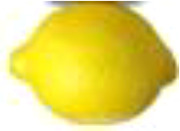	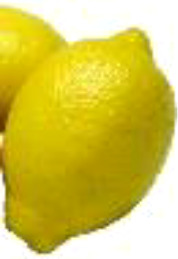	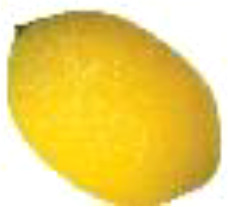	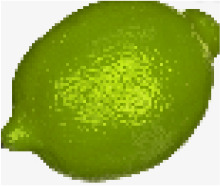	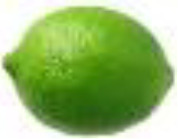
Peach	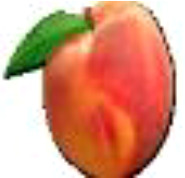	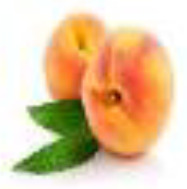	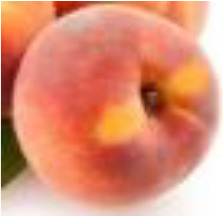	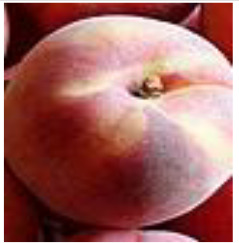	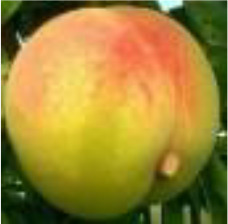	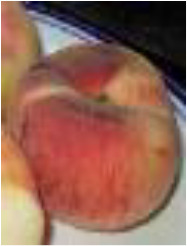	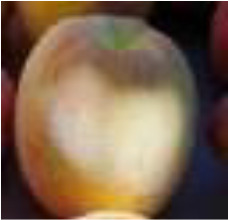	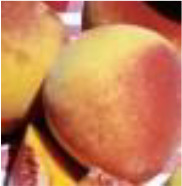	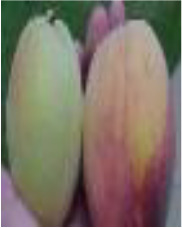	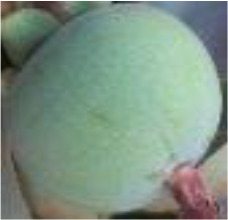	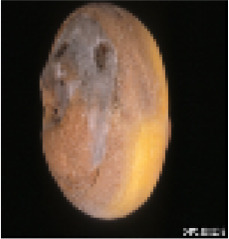	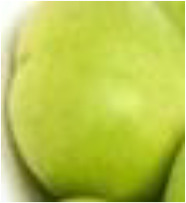

According to the objective, samples of tomato, lemon, red apple and peach have been selected to check the performance of the system. The default number of coloring points is 15, the upper threshold value is 185 and the lower threshold is 0, and the number of data used to obtain the average and standard deviation values is 10. In addition, in the table below, the examples used are given briefly.

## 4 Result and discussion

In this section, different examples are evaluated. Each of the samples has 3 extracted features. Each feature will be reviewed separately. Based on these extracted characteristics, each of the samples are placed in the category according to their quality. As a result, the reasons for the occurrence of errors and the number of errors, finally, solutions to improve the results of the method are examined.

In the case of tomatoes, the amount of color, size and weight changes according to the quality rating. Color is more important than size and weight in this case, red color plays a more important role in tomato samples. Of course, in the case of dehydration of the tomato tissue, the weight can indicate a decrease in the quality level. As the red color decreases, the quality also decreases. Finally, in the last degree, there is a possibility that green color is more than red. By taking a sample of red apples and comparing the characteristics, we can reach this conclusion, considering the assumption that defective fruits are not included in the classification system. The color clearly does not have that much effect on determining the quality level of the apple, the quality of the red apple is based on its weight and size, because according to the **Table 1** above, the smallest apple in the samples also has a dominant red color over green and blue. The closer the color is to zero, the darker the color of the apple. This can be a sign of excessive handling. Of course, too much darkness is not suitable. Too bright color can also be a sign that the apple is unripe. If the color level is closer to 255, the color of the apple is brighter, so both modes reduce the quality level. In some cases, the ratio of size and weight is not suitable compared to other cases. For example, the size has increased, but the weight has decreased. This case can be a sign of dehydration and apple texture. Lemon fruit in the first and second grade has a light color (light orange and yellow). In addition, the background color is light gray. In this situation, the measurement becomes problematic. Because the edges are not detected correctly. At the same time, large size and high weight in the first two grades help to distinguish the quality grade. Therefore, taking the size to some extent has an error, and in this situation, the weight is helpful.

### 4.1 Error reasons

In the conducted surveys, several reasons cause errors in sampling. 1- Measuring any amount that the color of the product tends to be bright, considering that the system makes the image binary for measurement, it becomes difficult to distinguish the white background from the product with a bright color. The edge is not accurately detected. Therefore, the size also has an error. 2- Changing the ambient light, in addition to causing errors in the amount of color calculation. In some cases, it also causes measurement errors. Its effect is that if there is white light, due to the white background of the weight sensor, edge detection becomes difficult. 3- The system of the system that executes the MATLAB program, if it has a delay, the commands are executed with a delay. It is better to use a robust system for implementation to minimize delays. 4- Webcams have low or high noise depending on the quality of construction. This amount will not be zero. Therefore, camera noise is effective in feature recognition. One of the things that affect is the number of separated colors.

### 4.2 Accuracy value

**Figure 3:**
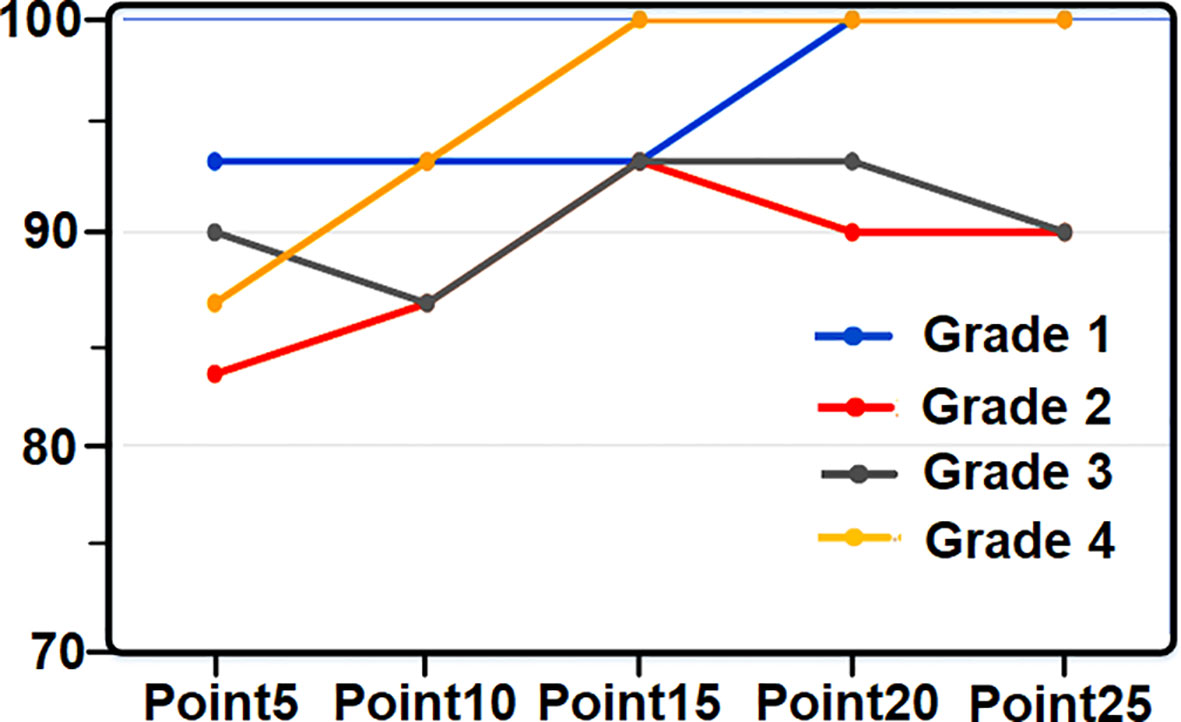
Tomato grade recognition chart based on coloring points.

To get the error rate, the difference amount is calculated by changing two components, the number of points and the threshold. The results are obtained by changing the number of points on the amount of color and changing the threshold. Using another example, the following table which is the value of the Confusion matrix is obtained. In **Table 2**, the accuracy of changing the coloring points of tomatoes has been specified. The diagram of tomato grade recognition based on coloring points is also shown in **Figure 3**.

**Table 2:** Accuracy with changing the coloring points of tomatoes.

Number of points	Grade 1 Accuracy	Grade 2 Accuracy	Grade 3 Accuracy	Grade 4 Accuracy
5	28	26	27	28
10	28	25	25	29
15	29	28	28	30
20	30	29	28	30
25	30	29	28	30

**Figure 4:**
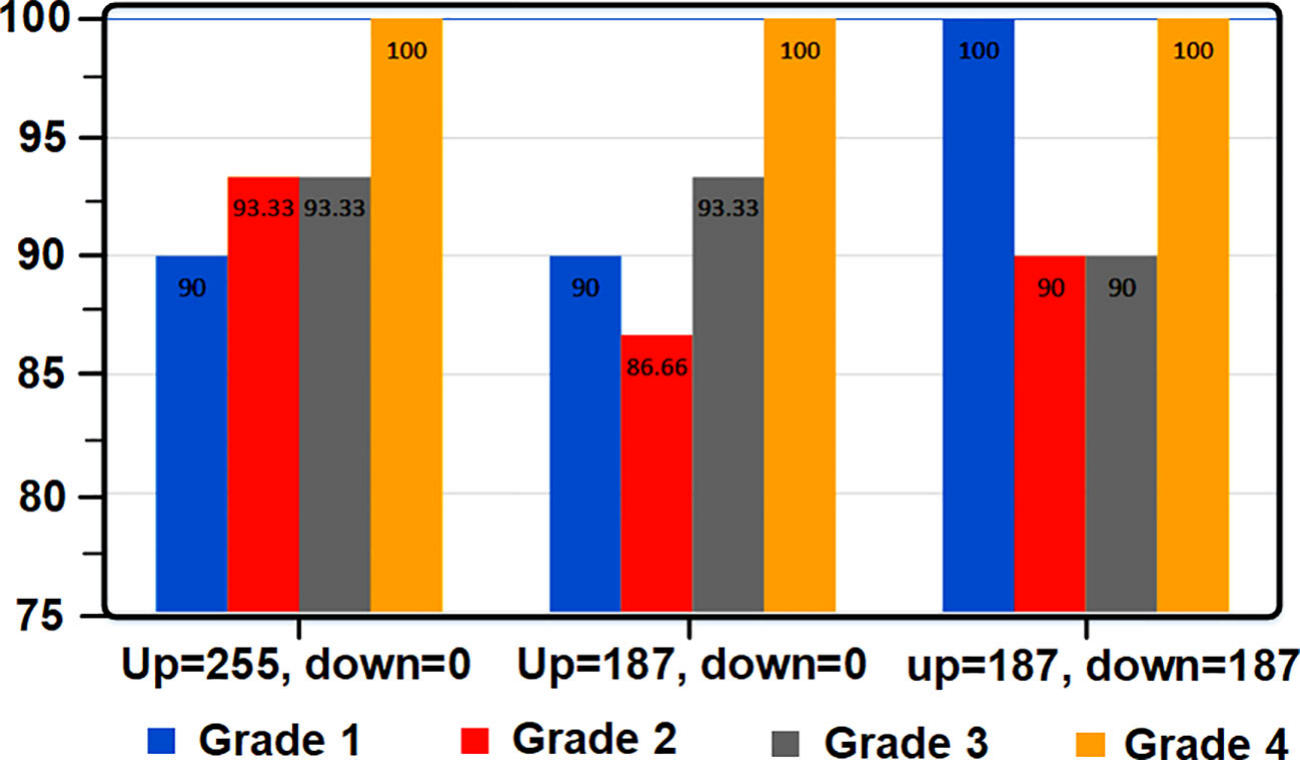
Diagram of tomato grade detection percentage based on threshold level changes.

**Table 3** shows the accuracy values with the change of the tomato threshold, **Figure 4** shows the percentage of tomato grade recognition based on the changes of the threshold level. The values of tomatoes are listed in **Table 4**.

**Table 3:** Accuracy by changing the tomato threshold.

Threshold rate	Grade 1 Accuracy	Grade 2 Accuracy	Grade 3 Accuracy	Grade 4 Accuracy
Up=255, down=0	29	28	28	30
Up=187, down=0	29	27	28	30
Up=187, down=187	30	29	29	30

**Table 4:** Tomato clutter table.

Real class	Anticipated class	Grade 1	Grade 2	Grade 3	Grade 4
Grade 1		29	1	0	0
Grade 2		1	28	1	0
Grade 3		0	1	28	1
Grade 4		0	0	1	29

**Figure 5:**
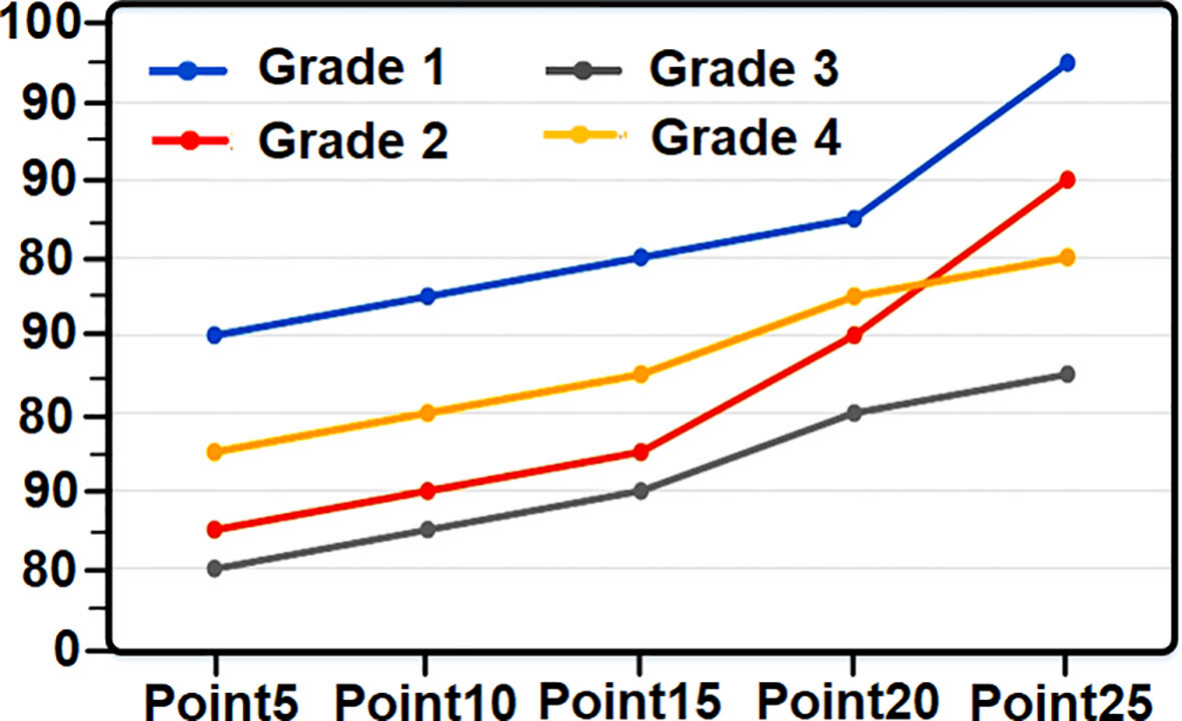
Apple grade recognition chart based on coloring points.

The results obtained for tomatoes show that the accuracy rate for each level of 30 samples (120 in total) is equal to 93.33%. This level of accuracy is due to the simultaneous decrease in color, size and weight with the lowering of the quality level. **Table 5** shows the accuracy values by changing the coloring points of apples. **Figure 5** also shows the apple grade recognition chart based on coloring points.

**Table 5:** Accuracy by changing the color points of the apple.

Number of points	Grade 1 Accuracy	Grade 2 Accuracy	Grade 3 Accuracy	Grade 4 Accuracy
5	32	28	24	28
10	24	24	28	10
15	32	28	28	28
20	32	32	28	32
25	40	36	28	32

**Figure 6:**
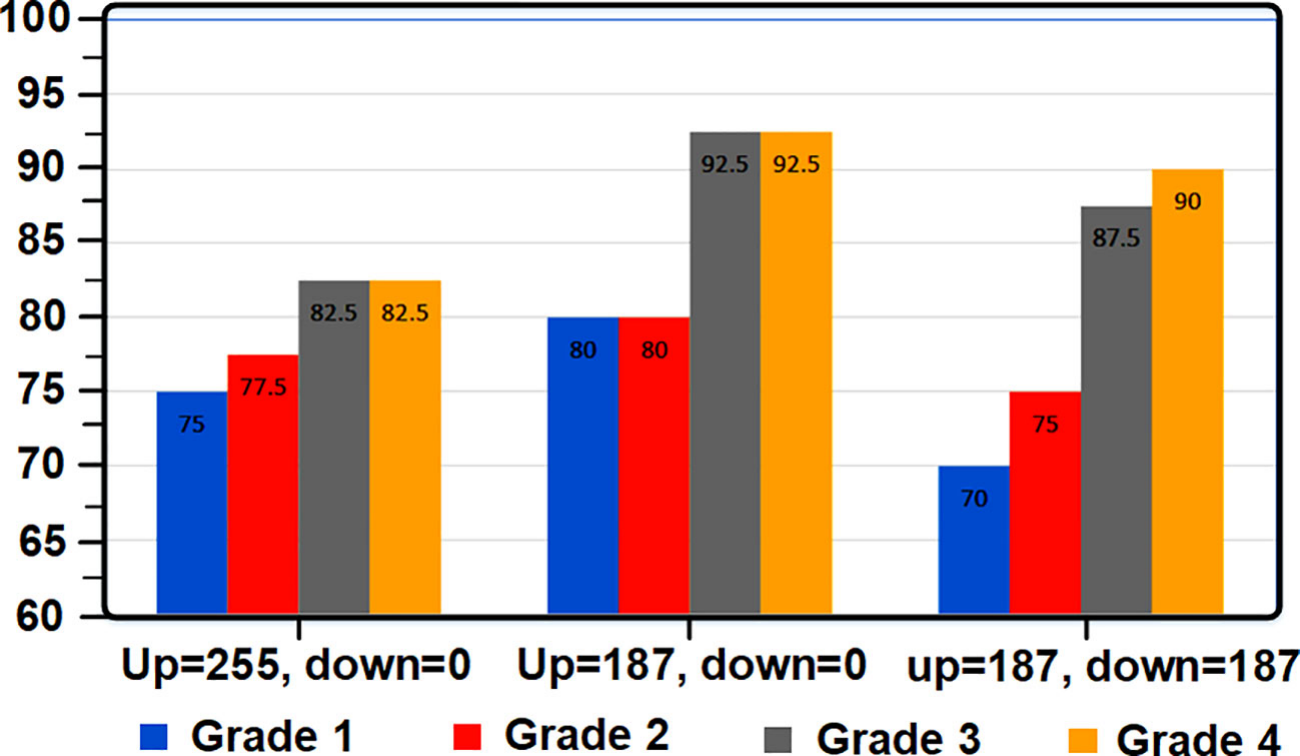
Apple grade recognition percentage chart based on threshold level changes.

**Table 6** shows the accuracy by changing the apple threshold. **Figure 6** also shows the apple grade recognition percentage graph based on changes in the threshold level, and the apple confusion table is specified in **Table 7**.

**Table 6:** Accuracy by changing apple threshold.

Threshold rate	Grade 1 Accuracy	Grade 2 Accuracy	Grade 3 Accuracy	Grade 4 Accuracy
Up=255, down=0	28	28	32	32
Up=187, down=0	32	32	36	36
Up=187, down=187	28	28	36	36

**Table 7:** Table of apple confusion.

Real class	Anticipated class	Grade 1	Grade 2	Grade 3	Grade 4
Grade 1		32	8	0	0
Grade 2		4	28	4	4
Grade 3		0	4	28	8
Grade 4		0	4	8	28

The values in the above tables show the amount of size difference by changing the threshold value and increasing the number of coloring points for apple fruit which is considered the original sample. Changing the upper and lower threshold value has an effect on the number of points counted in the background and fruit area. Changing the points affects the color analysis. According to the above values, the minimum accuracy is equal to 28 and the maximum accuracy is equal to 32. Roughly, the minimum accuracy is 70% and the maximum is 90%. The working time of the system for each processing is two seconds and a total of 160 samples and 320 seconds.

**Figure 7:**
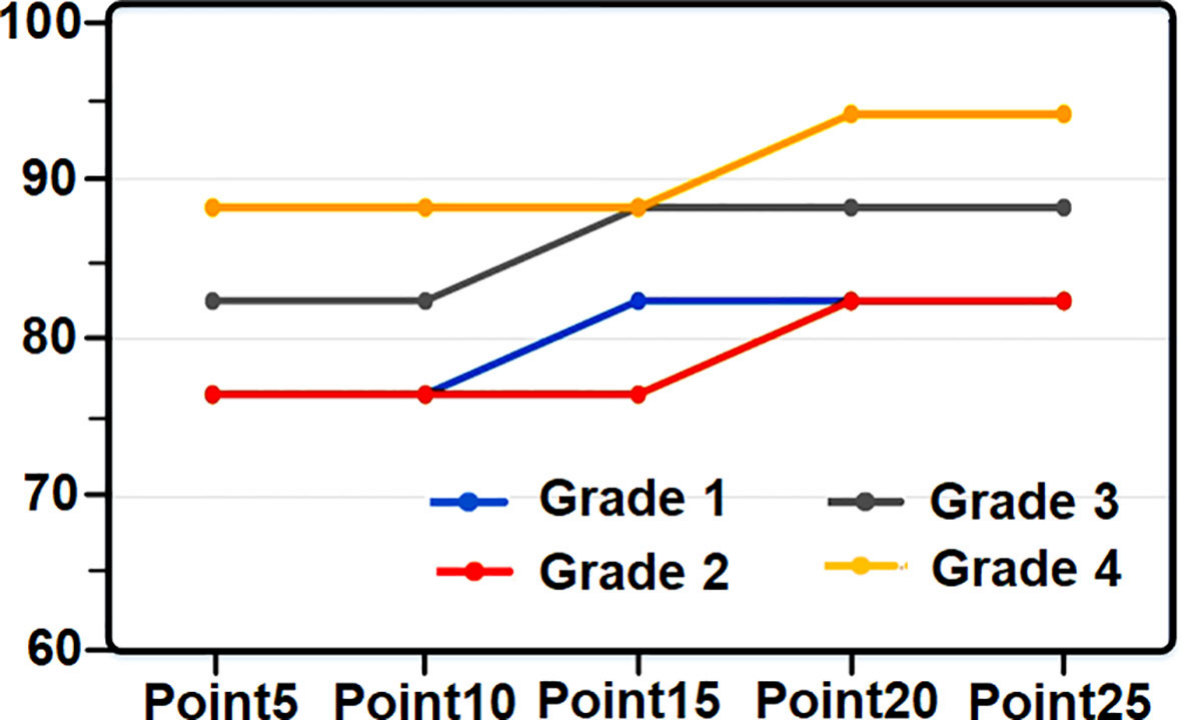
Diagram of recognition of lemon grade based on coloring points.

To compare the performance of the accuracy rate, the detection rate of tomato and lemon grades has been calculated. **Table 8** shows the accuracy values by changing lemon coloring points, and the diagram of lemon grade recognition based on coloring points is shown in **Figure 7**.

**Table 8:** Accuracy by changing the coloring points of the lemon.

Number of points	Grade 1 Accuracy	Grade 2 Accuracy	Grade 3 Accuracy	Grade 4 Accuracy
5	13	13	14	15
10	13	13	14	15
15	14	13	15	15
20	14	14	15	16
25	14	14	15	16

**Figure 8:**
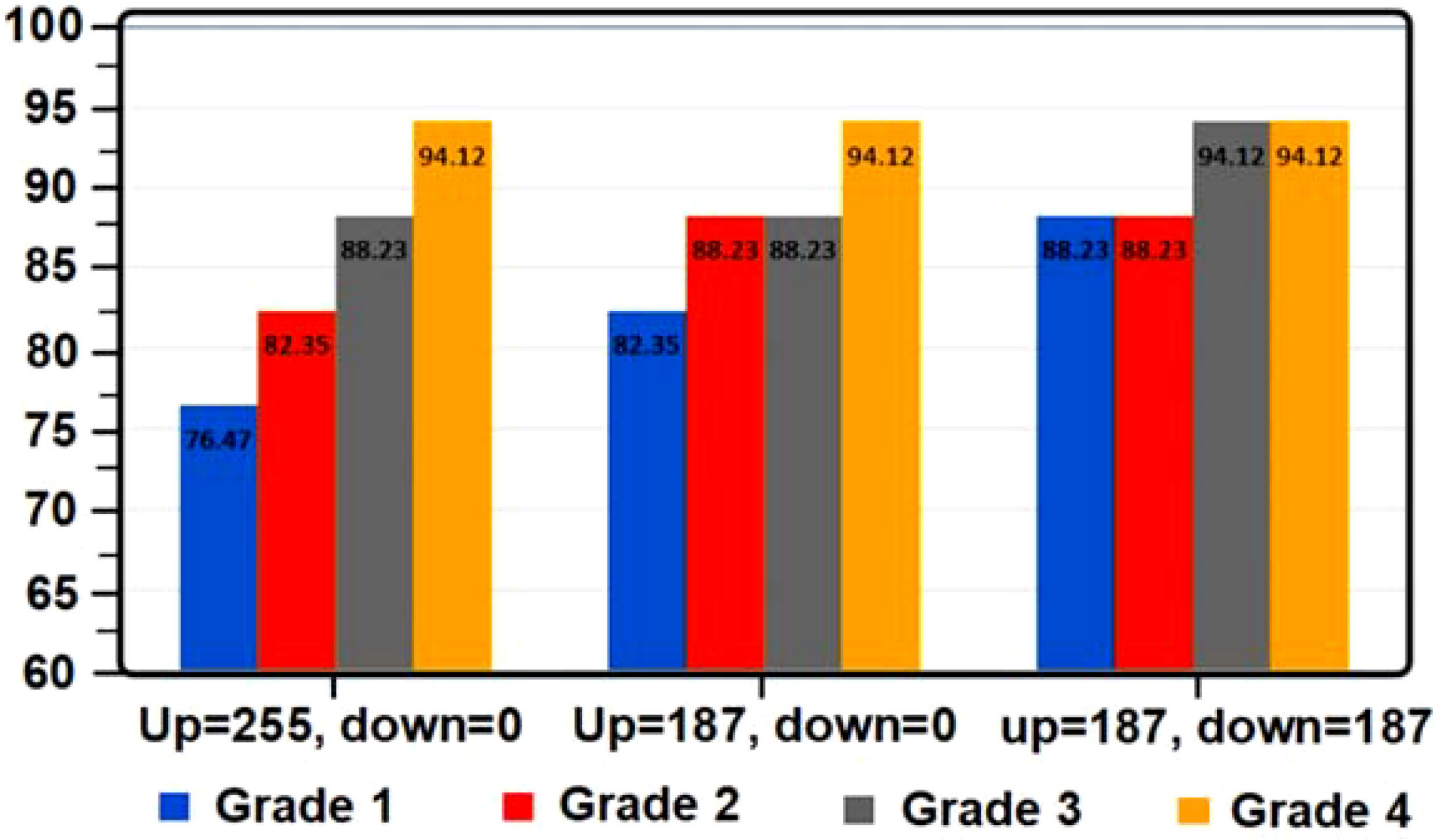
Diagram of lemon grade recognition percentage based on threshold level changes.

**Table 9** shows the accuracy values by changing the lemon threshold. **Figure 8** shows the lemon grade detection percentage graph based on changes in the threshold level. The table of lemon breakdown is given in **Table 10**.

**Table 9:** Accuracy by changing the lemon threshold. .

Threshold rate	Grade 1 Accuracy	Grade 2 Accuracy	Grade 3 Accuracy	Grade 4 Accuracy
Up=255, down=0	13	14	15	16
Up=187, down=0	14	15	15	16
Up=187, down=187	15	15	16	16

**Table 10:** Table of lemon confusion.

Real class	Anticipated class	Grade 1	Grade 2	Grade 3	Grade 4
Grade 1		15	2	0	0
Grade 2		1	15	1	0
Grade 3		0	1	15	1
Grade 4		0	0	0	17

**Figure 9:**
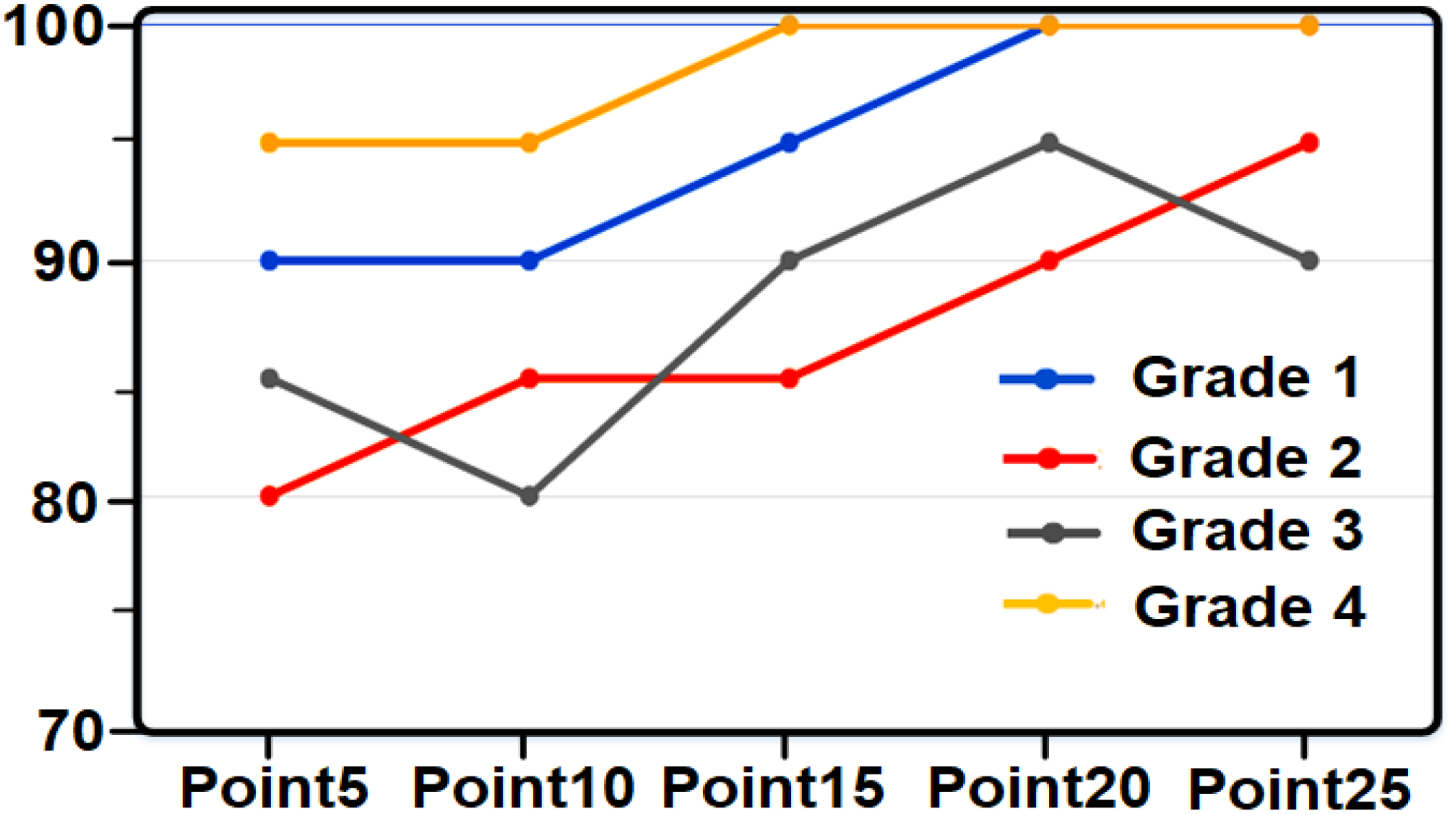
Peach grade detection chart based on coloring points.

The above table is based on measuring the color, size, and weight of 68 lemon samples. The accuracy rate is 88.23%. The accuracy rate has decreased compared to the tomato accuracy. The reason for that is the first and second-grade light color. **Table 11** shows the accuracy of changing the coloring points of peaches. **Figure 9** shows the diagram of peach grade recognition based on coloring points.

**Table 11:** Accuracy by changing the peach coloring points.

Number of points	Grade 1 Accuracy	Grade 2 Accuracy	Grade 3 Accuracy	Grade 4 Accuracy
5	18	16	17	19
10	18	17	16	19
15	19	17	18	20
20	20	18	19	20
25	20	19	18	20

**Figure 10:**
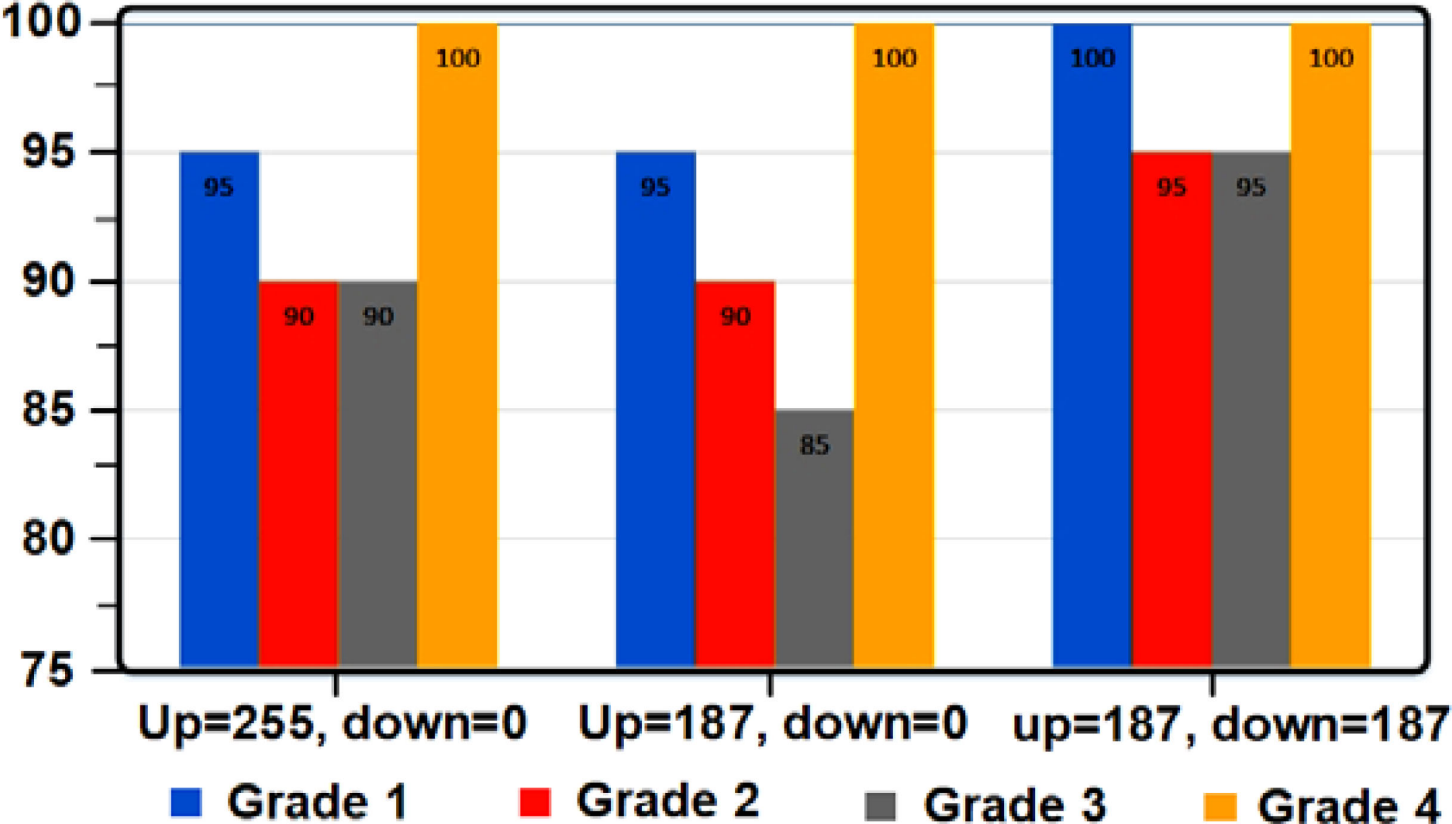
Diagram of the detection percentage of the peach degree based on the threshold level changes.

**Table 12** shows the accuracy values with the change of the peach threshold. **Figure 10** shows the percentage of peach grade detection based on changes in the threshold level, and the table of peach breakdown is also given in **Table 13**.

**Table 12:** Accuracy by changing the peach threshold.

Threshold rate	Grade 1 Accuracy	Grade 2 Accuracy	Grade 3 Accuracy	Grade 4 Accuracy
Up=255, down=0	19	18	18	20
Up=187, down=0	19	18	17	20
Up=187, down=187	20	19	19	20

**Table 13:** Table of peach confusion.

Real class	Anticipated class	Grade 1	Grade 2	Grade 3	Grade 4
Grade 1		18	2	0	0
Grade 2		4	16	0	0
Grade 3		0	1	17	2
Grade 4		0	0	0	20

**Figure 11:**
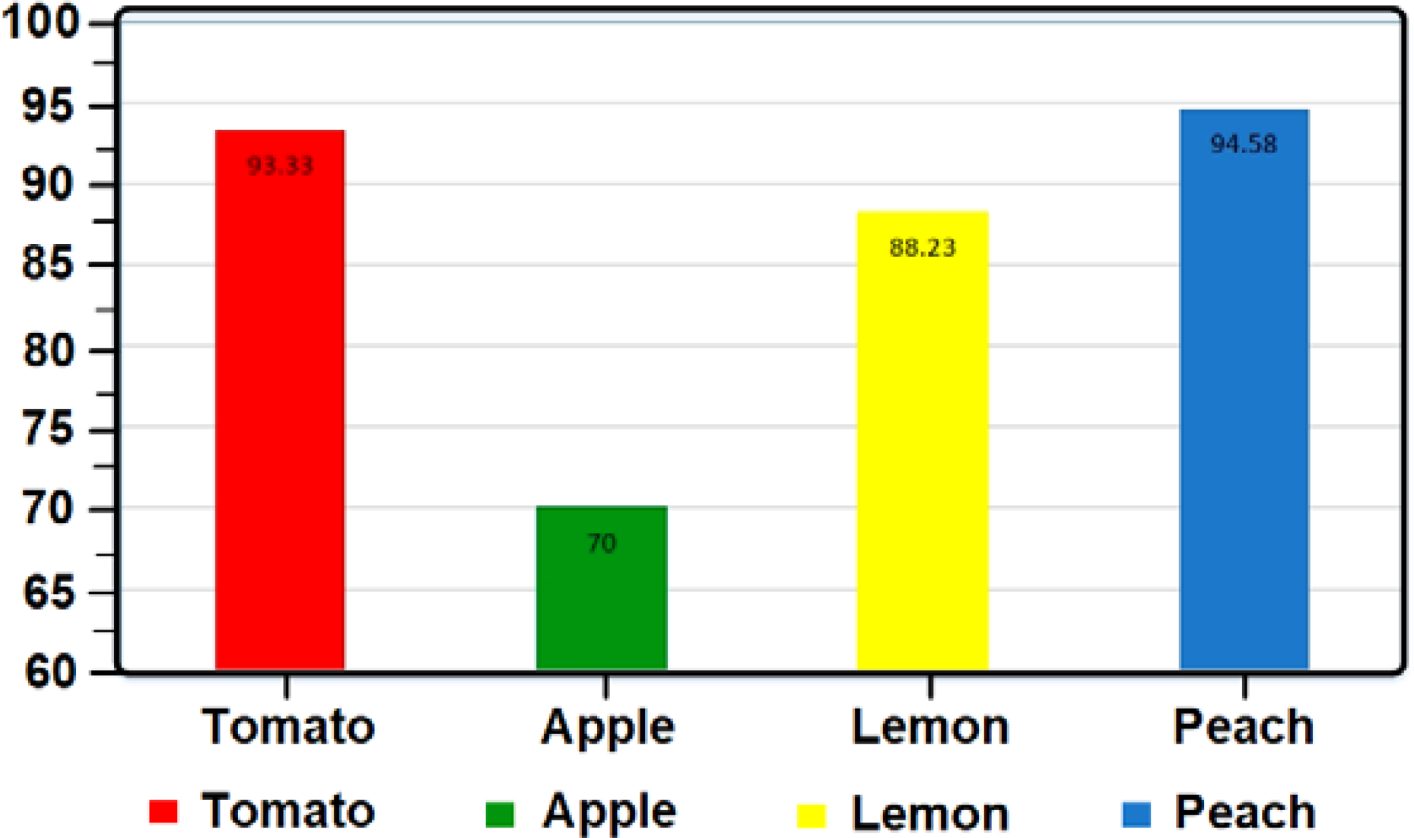
Accuracy diagram.

The results obtained for peach show that the accuracy rate for each level of 20 samples is 94.58%. This level of accuracy is due to the simultaneous change of color, size, and weight. The diagram in **Figure 11** shows the final result of the 4 accuracy tables for each of the tested fruits.

As mentioned above, detecting the color and size of peach and tomato products due to their intense red color and significant changes of this color in different degrees has a higher percentage of accuracy. The solutions to reducing ambient light errors are 1- At a certain hour and under the same conditions, if the chamber is not used, the checks should be done. 2- The creation of shadows or white light in the laboratory environment is prevented. 3- A chamber with constant light should be used so that no light enters this chamber from the outside. In this case, both the color error and the measurement error are reduced. 4- The standard deviation of the measurements is created in the part of the program created in MATLAB. 5- The minimum and maximum values for all three items (color, size, weight) are considered.

According to the results, it can be said that the proposed method can be a useful method for the packaging industry. So that if desired, there is the ability to change the indicators based on the need, which is considered a major advantage. As it turns out, this is made under limited conditions and has the ability to improve.

## 5 Conclusion

Today, speed plays a very important role in the development of an industry. Therefore, it can be of great help to the industry of a country that is trying to increase its non-oil income by building automatic systems. This article presents statistical machine learning technologies with machine vision systems in agriculture due to the wide range of machine learning applications. The proposed method aims to build a classification system with low cost and time but with favorable results. MATLAB software has the ability to perform mathematical calculations and combine inputs. It is a powerful and efficient software for image processing. It can also be used in many specialized cases, especially those that require online time. Therefore, according to observations, the error rate is very low. In fact, the program itself does not have errors, but environmental conditions cause errors. With this method, the speed can be increased simultaneously with accuracy. Therefore, eye error will not play a role in the result. The highest level of accuracy for peach fruit is 94.58% for each level of 20 samples. By comparing the proposed method and recent works, the proposed method is more favorable and efficient than the previous methods. Regarding fruit classification, weight plays a more important role than color. As a result, the combination of weight with the two characteristics of color and size can increase the accuracy of product classification.

## Data Availability

The original contributions presented in the study are included in the article/**Supplementary Material**, further inquiries can be directed to the corresponding author/s.
