# Supplementary material for: Quality detection and grading of peach fruit based on image processing method and neural networks in agricultural industry
Source: Front Plant Sci. 2024 Sep 20;15:1415095. doi: 10.3389/fpls.2024.1415095 (PMC11452848; doi:10.3389/fpls.2024.1415095)
Supplement: Supplementary file 1 [file DataSheet1.docx]

Supplementary material

In MATLAB software, it takes the image from the camera (webcam) in real time using the function cam = videoinput ('winvideo'). At the same time, the front part of the image can be displayed in raw black and white. Using the output wire, the image is sent to the next section for analyzing the characteristics. Figure 1 shows the image of the camera.


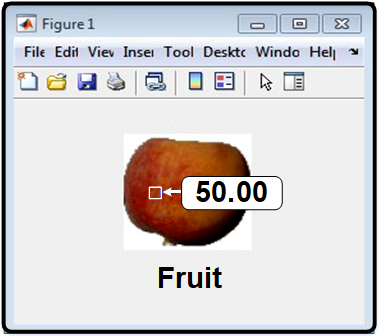


**Fig. 1.** Image is taken from the camera.

To measure color, as mentioned in the previous chapter, red, green, and blue colors are separated and their values ​​are measured separately. The method of getting the color is that you can get the amount of color from a pre-defined point. The steps of fruit color calculation are shown in Figure 2. But the experiments it shows that taking the color from several points and taking the average of all three colors red, green, and blue reduces the amount of error. Therefore, in this method, n points (minimum 5 and maximum 25) are used to measure color.


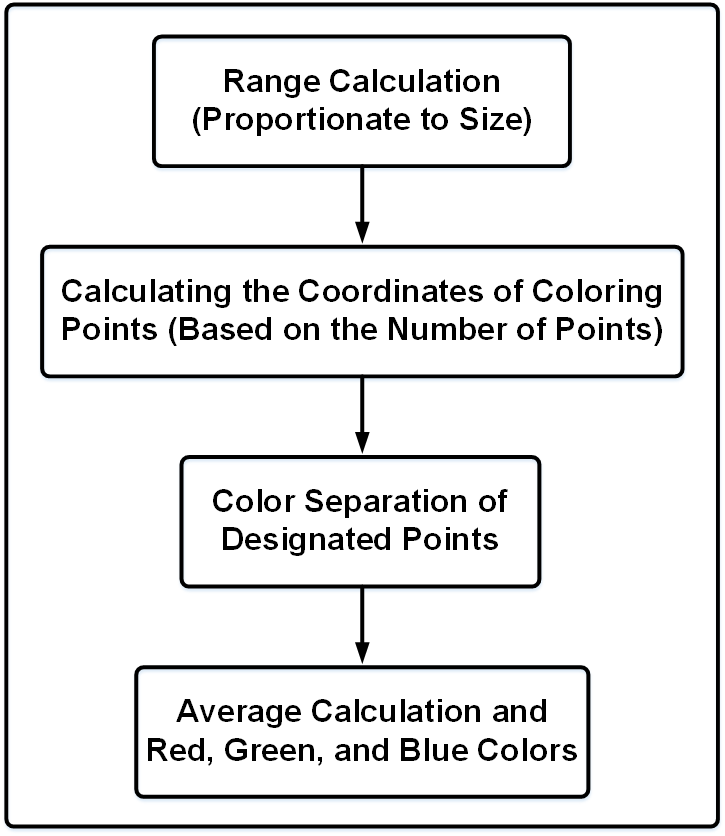


**Fig. 2.** Steps of fruit color calculation.

Figure 3 shows the range considered in peach fruit. Points are considered within the specified range.


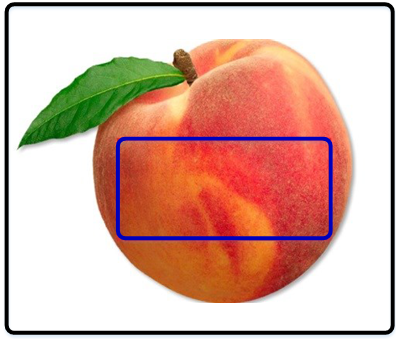


**Fig. 3.** Range considered.

In this method, a range is defined according to the size of the fruit. The number of points in this range is selected, finally, the average color and the average values ​​of red, green, and blue are obtained. The input image in this section has two RGB (32) and HSL (32) modes.

Figure 4 shows the measured sample and Figure 6 shows the color analysis sample.


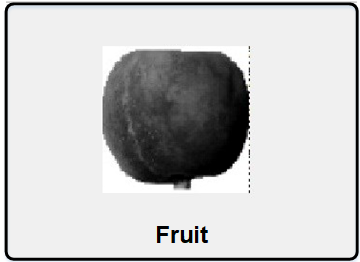


**Fig. 4.** Shows the measured sample.
